# Epidemiological features of primary glomerular disease in Turkey: a multicenter study by the Turkish Society of Nephrology Glomerular Diseases Working Group

**DOI:** 10.1186/s12882-020-02134-8

**Published:** 2020-11-14

**Authors:** Aydin Turkmen, Abdullah Sumnu, Egemen Cebeci, Halil Yazici, Necmi Eren, Nurhan Seyahi, Kamil Dilek, Fatih Dede, Ulver Derici, Abdulkadir Unsal, Garip Sahin, Murat Sipahioglu, Mahmut Gok, Erhan Tatar, Belda Dursun, Savas Sipahi, Murvet Yilmaz, Gultekin Suleymanlar, Sena Ulu, Ozkan Gungor, Sim Kutlay, Zerrin Bicik Bahcebasi, Idris Sahin, Ilhan Kurultak, Kultigin Turkmen, Zulfikar Yilmaz, Rumeyza Turan Kazancioglu, Caner Cavdar, Ferhan Candan, Zeki Aydin, Duriye Deren Oygar, Cuma Bulent Gul, Mustafa Arici, Saime Paydas, Dilek Guven Taymez, Mehmet Kucuk, Sinan Trablus, Kenan Turgutalp, Leyla Koc, Siren Sezer, Murat Duranay, Simge Bardak, Lutfullah Altintepe, Izzet Hakki Arikan, Alper Azak, Ali Riza Odabas, Gulizar Manga Sahin, Savas Ozturk

**Affiliations:** 1 Istanbul Medical Faculty, Nephrology, Istanbul University, Istanbul, Turkey; 2 Medical Faculty, Nephrology, Istanbul Medipol University, Istanbul, Turkey; 3 Haseki Training and Research Hospital, Nephrology, Istanbul, Turkey; 4 Medical Faculty, Nephrology, Kocaeli University, Kocaeli, Turkey; 5 Cerrahpasa Medical Faculty, Nephrology, Istanbul University, Istanbul, Turkey; 6 Medical Faculty, Nephrology, Uludag University, Bursa, Turkey; 7 Ankara Numune Training and Research Hospital, Nephrology, Ankara, Turkey; 8 Medical Faculty, Nephrology, Gazi University, Ankara, Turkey; 9 Hamidiye Sisli Etfal Training and Research Hospital, Nephrology, Istanbul, Turkey; 10 Medical Faculty, Nephrology, Eskisehir Osmangazi University, Eskisehir, Turkey; 11 Medical Faculty, Nephrology, Erciyes University, Kayseri, Turkey; 12 Sultan Abdulhamit Han Training and Research Hospital, Nephrology, Istanbul, Turkey; 13 Izmir Bozyaka Training and Research Hospital, Nephrology, Izmır, Turkey; 14 Medical Faculty, Nephrology, Pamukkale University, Denizli, Turkey; 15 Medical Faculty, Nephrology, Sakarya University, Adapazari, Sakarya Turkey; 16 Bakirkoy Sadi Konuk Training and Research Hospital, Nephrology, Istanbul, Turkey; 17 Medical Faculty, Nephrology, Akdeniz University, Antalya, Turkey; 18 Medical Faculty, Nephrology, Afyonkarahisar University, Afyon, Turkey; 19 Medical Faculty, Nephrology, Sutcu İmam University, Kahramanmaras, Turkey; 20 Medical Faculty, İbni Sina Hospital, Nephrology, Ankara University, Ankara, Turkey; 21 Dr. Lutfi Kirdar Kartal Training and Research Hospital, Nephrology, Istanbul, Turkey; 22 Medical Faculty, Nephrology, Inonu University, Malatya, Turkey; 23 Medical Faculty, Nephrology, Trakya University, Edirne, Turkey; 24 Meram Medical Faculty, Nephrology, Necmettin Erbakan University, Konya, Turkey; 25 Medical Faculty, Nephrology, Dicle University, Diyarbakir, Turkey; 26 Medical Faculty, Nephrology, Bezmialem Vakif University, Istanbul, Turkey; 27 Medical Faculty, Nephrology, Dokuz Eylul University, Izmir, Turkey; 28 Medical Faculty, Nephrology, Cumhuriyet University, Sivas, Turkey; 29 Darica Farabi Training and Research Hospital, Nephrology, Kocaeli, Turkey; 30 Doktor Burhan Nalbantoglu State Hospital, Lefkosa, Cyprus; 31 Bursa Yuksek Ihtisas Training and Research Hospital, Nephrology, Bursa, Turkey; 32 Medical Faculty, Nephrology, Hacettepe University, Ankara, Turkey; 33 Medical Faculty, Nephrology, Cukurova University, Adana, Turkey; 34 Kocaeli State Hospital, Nephrology, Kocaeli, Turkey; 35 Okmeydani Training and Research Hospital, Nephrology, Istanbul, Turkey; 36 Istanbul Training and Research Hospital, Nephrology, Istanbul, Turkey; 37 Medical Faculty, Nephrology, Mersin University, Mersin, Turkey; 38 Taksim Training and Research Hospital, Nephrology, Istanbul, Turkey; 39 Medical Faculty, Nephrology, Baskent University, Ankara, Turkey; 40 Ankara Training and Research Hospital, Nephrology, Ankara, Turkey; 41 Batman State Hospital, Nephrology, Batman, Turkey; 42 Selcuk Medical Faculty, Nephrology, Selcuk University, Konya, Turkey; 43 Marmara University, Medical Faculty, Nephrology, Istanbul, Turkey; 44 Balikesir Training and Research Hospital, Nephrology, Balikesir, Turkey; 45 Goztepe Training and Research Hospital, Nephrology, Istanbul, Turkey

**Keywords:** Epidemiology, Glomerulonephritis, Kidney biopsy, Primary glomerular diseases; the Turkish Society of Nephrology glomerular diseases (TSN-GOLD) working group, Turkish Society of Nephrology

## Abstract

**Background:**

The largest data on the epidemiology of primary glomerular diseases (PGDs) are obtained from the databases of countries or centers. Here, we present the extended results of the Primary Glomerular Diseases Study of the Turkish Society of Nephrology Glomerular Diseases (TSN-GOLD) Working Group.

**Methods:**

Data of patients who underwent renal biopsy and received the diagnosis of PGD were recorded in the database prepared for the study. A total of 4399 patients from 47 centers were evaluated between May 2009 and May 2019. The data obtained at the time of kidney biopsy were analyzed. After the exclusion of patients without light microscopy and immunofluorescence microscopy findings, a total of 3875 patients were included in the study.

**Results:**

The mean age was 41.5 ± 14.9 years. 1690 patients were female (43.6%) and 2185 (56.3%) were male. Nephrotic syndrome was the most common biopsy indication (51.7%). This was followed by asymptomatic urinary abnormalities (18.3%) and nephritic syndrome (17.8%). The most common PGD was IgA nephropathy (25.7%) followed by membranous nephropathy (25.6%) and focal segmental glomerulosclerosis (21.9%). The mean total number of glomeruli per biopsy was 17 ± 10. The mean baseline systolic blood pressure was 130 ± 20 mmHg and diastolic blood pressure was 81 ± 12 mmHg. The median proteinuria, serum creatinine, estimated GFR, and mean albumin values were 3300 (IQR: 1467–6307) mg/day, 1.0 (IQR: 0.7–1.6) mg/dL, 82.9 (IQR: 47.0–113.0) mL/min and 3.2 ± 0.9 g/dL, respectively.

**Conclusions:**

The distribution of PGDs in Turkey has become similar to that in other European countries. IgA nephropathy diagnosed via renal biopsy has become more prevalent compared to membranous nephropathy.

## Background

Primary glomerular diseases (PGDs) are among the leading causes of chronic kidney disease (CKD) and end-stage renal disease (ESRD) both in Turkey and in the world. According to the registry data of Turkey, Europe, and the United States, PGDs are the third most common cause of ESRD after diabetes mellitus and hypertension among patients starting dialysis. According to the Turkish Society of Nephrology (TSN) 2017 annual registry report, the incidence of PGDs in patients starting hemodialysis and peritoneal dialysis was 6.01 and 12.27%, respectively [1]. In the European Dialysis and Transplant Association (ERA-EDTA) 2017 annual report, the incidence of PGDs in patients receiving renal replacement therapy was 6.3–20.0% [2]. Similarly, in the United States Renal Data System (USRDS) 2016 annual report, the prevalence of PGDs among patients starting renal replacement therapy was 8.6%, and this rate did not change significantly in recent years [3].

On the other hand, since PGDs are potentially preventable or treatable diseases, several research studies are being done to understand and ultimately treat these diseases. Additionally, PGDs are heterogeneous diseases that may show different clinical pictures at different ages, the course of the diseases usually lasts for many years and, their relative rarity makes it difficult to conduct studies on the epidemiology of these diseases at a single center. Although the epidemiological studies examining the prevalence, geographic distribution, and disease trends of PDGs are an important part of these researches, such studies are still scarce. Therefore, national glomerulonephritis (GN) registry systems, including epidemiological data, have been established.

Epidemiological data on PGDs vary between countries due to geographical, genetic and environmental differences as well as changes in medical approach, indications for biopsy, the prevalence of GNs, etc. For example, it has been reported that the incidence of membranous nephropathy (MN) has increased in China in recent years due to increased air pollution [4]. Therefore, data-based studies from different regions of the world can contribute to the assessment and management of PGDs.

We published the result of a study, including 1274 biopsy-proven PGD patients, performed by the TSN Glomerular Diseases (TSN-GOLD) Working Group in 2014 [5]. The most frequent PGD was MN (28.8%) followed by focal segmental glomerulosclerosis (FSGS) (19.3%) and IgA nephropathy (IgAN) (17.2%). This study investigated the changes in the presentation and frequency of PGDs and indications for biopsy in our country in the last 10 years.

## Methods

This multicenter cross-sectional study was conducted using data from a web-based database formed by the TSN-GOLD Working Group. At the time of the study, there were 60 authorized nephrology clinics in our country. Among these, 47 centers were included in the TSN-GOLD Group. Only data on patients with PGDs older than 16 years of age are entered into this database. Secondary glomerular diseases are not recorded in this system. IgAN, MN, FSGS, minimal change disease (MCD), membranoproliferative glomerulonephritis (MPGN), and crescentic glomerulonephritis (CGN) were accepted as the main PGD. The remaining less common PGDs were classified as “others”.

The database was prepared mostly using multiple-choice questions for standardized data. The demographic and clinical characteristics of the patients, in addition to the biochemical and histopathological findings, were asked. Ethical approval was obtained from The Ethics Committee of Istanbul University, Istanbul Medical Faculty.

In this study, only data collected during renal biopsy were included. Data from the follow-up period after biopsy were not included in the study. Non-diagnostic biopsy samples were excluded. Biopsy specimens were examined in the pathology laboratory of each center. Demographic parameters, history of chronic diseases such as diabetes mellitus, hypertension, and cardiovascular disease, indication for renal biopsy, pathological diagnosis, the department at which the biopsy was carried out, and comprehensive description of pathological findings were recorded in the database. The biopsy indications were classified as asymptomatic urinary abnormalities (AUA), nephrotic syndrome, nephritic syndrome, including rapidly progressive glomerulonephritis (RPGN), mixed nephrotic syndrome, and others. Persistent non-nephrotic proteinuria and/or isolated microscopic hematuria were defined as AUA. Nephrotic syndrome was defined by the presence of proteinuria (protein excretion > 3.5 g/24 h) along with hypoalbuminemia, edema, and hyperlipidemia. Nephritic syndrome was defined by hematuria, proteinuria (< 3.5 g/day), hypertension and decreased glomerular filtration rate (GFR). Mixed nephrotic syndrome was defined by nephrotic syndrome comprising findings of nephritic syndrome. RPGN was defined by a rapid decrease in GFR within days or weeks associated with any GN. Estimated GFR (eGFR) was calculated using the Chronic Kidney Disease Epidemiology Collaboration (CKD-EPI) equation [6].

The Statistical Package for the Social Sciences version 17.0 for Windows (SPSS Inc., Chicago, IL, USA) was used for statistical analyses. Normally-distributed variables were presented as percent or mean ± standard deviation and non-normally distributed variables as median and interquartile range.

## Results

**Fig. 1:**
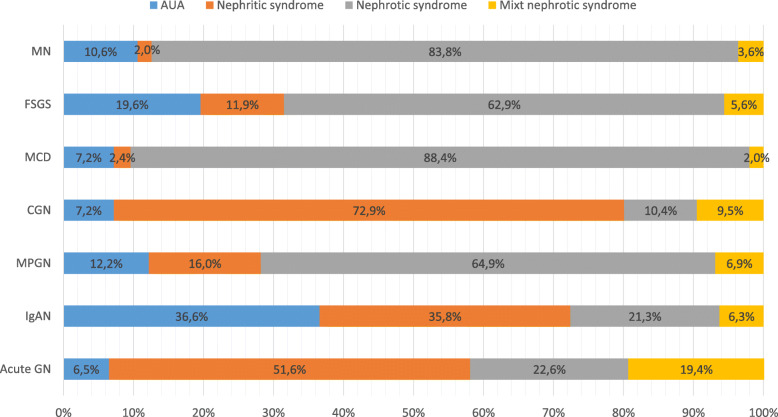
Indications for renal biopsy according to the PGDs. (Abbreviations: Acute GN: acute glomerulonephritis, AUA: Asymptomatic urinary abnormalities, CGN: crescentic glomerulonephritis, FSGS: Focal segmental glomerulosclerosis, IgAN: IgA nephropathy, MCD: minimal change disease, MN: membranous nephropathy, MPGN: membranoproliferative glomerulonephritis)

**Fig. 2:**
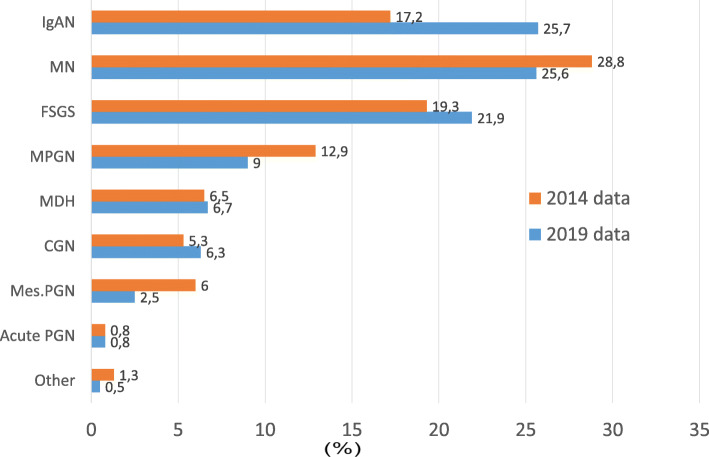
Diagnosis of patients according to the renal biopsy results. The data are presented separately for each PDG based on the current study and the data of our group published in 2014 [5]. (Abbreviations: Acute PGN: acute proliferative glomerulonephritis CGN: crescentic glomerulonephritis, FSGS: Focal segmental glomerulosclerosis, IgAN: IgA nephropathy, MCD: minimal change disease, Mes.PGN: Mesangioproliferative glomerulonephritis (non-IgA), MN: membranous nephropathy, MPGN: membranoproliferative glomerulonephritis)

**Fig. 3:**
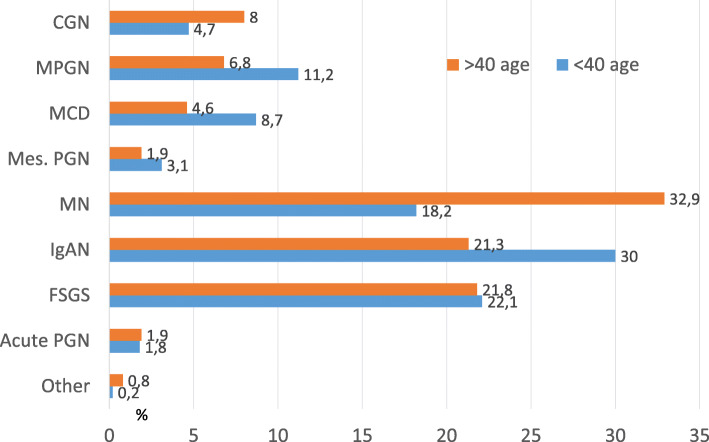
Distribution of PDG in age groups < 40 and > 40 years. (Abbreviations: Acute PGN: acute proliferative glomerulonephritis CGN: crescentic glomerulonephritis, FSGS: Focal segmental glomerulosclerosis, IgAN: IgA nephropathy, MCD: minimal change disease, Mes.PGN: Mesangioproliferative glomerulonephritis (non-IgA), MN: membranous nephropathy, MPGN: membranoproliferative glomerulonephritis)

A total of 4399 patients from 47 centers recorded between May 2009 and May 2019 were evaluated. Data from a total of 3875 patients were analyzed after the exclusion of patients without light microscopy and immunofluorescence microscopy (IF) findings. 1690 patients were female (43.6%) and 2185 (56.4%) were male. Nephrotic syndrome was the most common indication for renal biopsy (52.7%) followed by AUA (18.7%) and nephritic syndrome (18.2%). Figure 1 shows the distribution of renal biopsy indications according to the PGDs. The most common PGD was IgAN (25.7%) followed by MN (25.6%) and FSGS (21.9%). Figure 2 shows the distribution of PGDs in our current data and in the previous data published in 2014. While the incidence of IgAN and FSGS increased, MN, MPGN and mesangioproliferative GN were found to be decreased. There was no significant change in the frequency of MCD. When the patients were divided into two age groups (Fig. 3), the leading etiology was MN in patients aged > 40 years and IgAN in those aged < 40 years. There was no difference in FSGS between these age groups. However, MCD and MPGN were almost twice frequent in patients under 40 years of age compared to those over 40. On the other hand, CGN was almost twice frequent in patients over 40 years of age than in those under 40.

Patients’ data obtained during kidney biopsy are presented in Table 1. The data of patients, according to the PGD type, are presented in Table 2. The mean baseline systolic and diastolic blood pressure were 130 ± 20 mmHg and 81 ± 12 mmHg, respectively. About half of the patients (1722 of 3456 patients, 49.8%) had edema at presentation. The median proteinuria was 3300 (IQR: 1467–6307) mg/day, mean serum creatinine, eGFR and albumin values were 1.4 ± 1.5 mg/dL, 80.7 ± 39.1 mL/min/m^2^ and 3.2 ± 0.9 g/dL, respectively. Medical history revealed type 1 diabetes mellitus in 0.26% (10 of 3730 patients), type 2 diabetes mellitus 8.7% (327 of 3730 patients), and hypertension in 32.4% (1213 of 3735 patients) of the patients. About 33.7% of the patients (1308 of 3875 patients) were using a renin-angiotensin-aldosterone-system blocker before the renal biopsy.

**Table 1:** Patients’ data at the time when kidney biopsy was performed

	Mean ± SD
Systolic blood pressure (mmHg)	130 ± 20
Diastolic blood pressure (mmHg)	81 ± 12
BMI (kg/m^2^)	27.3 ± 9.7
Glucose (mg/dL)	97 ± 31
BUN (mg/dL)	23 ± 19
Creatinine (mg/dL)^a^	1.0 (0.7–1.6)
eGFR (CKD-EPI, mL/min/1.73m^2^)^a^	82.9 (47.0–113.0)
The total number of glomeruli (per biopsy)	17 ± 10
Uric acid (mg/dL)	6.2 ± 1.8
Triglyceride (mg/dL)^a^	175.5 (121.0–256.0)
LDL-cholesterol (mg/dL)	163 ± 85
ALT (IU/L)^a^	17 (13.0–24.0)
Calcium (mg/dL)	8.8 ± 0.8
Hemoglobin (g/dL)	12.9 ± 2.1
Serum albumin (g/dL)	3.2 ± 0.9
Proteinuria (mg/day)^a^	3300 (1467–6307)

*BMI* Body mass index, *eGFR* Estimated glomerular filtration rate. ^a^Median (interquartile range)

**Table 2:** The data of patients according to the type of PGD

	MCD (***n*** = 259)	FSGS (***n*** = 848)	MN (***n*** = 992)	IgAN (***n*** = 995)	Mes. PGN (***n*** = 97)	MPGN (***n*** = 349)	Acute PGN (***n*** = 31)	CGN (***n*** = 244)
**Age** Mean ± SD	36.1 ± 15.4	40.4 ± 14.2	46.7 ± 14.5	38.4 ± 13	36.8 ± 13.8	37.3 ± 15.6	43.1 ± 17.2	48 ± 16.6
**Gender** n (%) Woman	118 (45.9)	415 (48.9)	409 (41.3)	371 (37.3)	56 (58.9)	168 (48.3)	13 (40.6)	112 (45.5)
**Creatinine** (mg /dL) Mean ± SD	0.89 ± 0.65	1.29 ± 1.17	1 ± 0.8	1.55 ± 1.26	0.97 ± 0.69	1.51 ± 1.41	2.23 ± 2.22	4.1 ± 3.34
**eGFR (CKD-EPI)** mL/min Mean ± SD	105 ± 33	82 ± 36	94 ± 33	72 ± 36	99 ± 33	77 ± 40	62 ± 43	30 ± 30
**Serum albumin** (g/dL) Mean ± SD	2.61 ± 1.03	3.39 ± 0.93	2.77 ± 0.86	3.85 ± 0.69	3.52 ± 0.9	3.15 ± 0.89	3.19 ± 0.85	3.24 ± 0.73
**Proteinuria** (mg/day) Median (IQR)	5835 (2400–9000)	3400 (1770–5740)	6000 (3400–9280)	1822 (1000–3400)	1600 (540–3900)	3500 (1400–6120)	2310 (900–7435)	2100 (1230–3690)
**Hematuria**n n (%)	47 (18.2)	277 (32.6)	288 (29.1)	633 (63.7)	42 (43.8)	170 (48.7)	22 (68.8)	191 (77.6)
**Pyuria**n (%)	31 (13.5)	113 (15.2)	115 (13.2)	178 (20.0)	15 (18.8)	83 (31.4)	8 (28.6)	92 (41.1)
**SBP (mmHg)** Mean ± SD	122 ± 17	131 ± 19	129 ± 19	130 ± 21	129 ± 25	132 ± 23	142 ± 23	136 ± 20
**DBP (mmHg)** Mean ± SD	76 ± 10	81 ± 11	81 ± 11	82 ± 12	81 ± 15	82 ± 13	84 ± 13	82 ± 11

*Abbreviations*: *Acute PGN* acute proliferative glomerulonephritis, *CGN* crescentic glomerulonephritis, *DPB* diastolic blood pressure, *eGFR* estimated glomerular filtration rate, *FSGS* Focal segmental glomerulosclerosis, *IgAN* IgA nephropathy, *MCD* minimal change disease, *Mes.PGN* Mesangioproliferative glomerulonephritis (non-IgA), *MN* membranous nephropathy, *MPGN* membranoproliferative glomerulonephritis, *SPB* systolic blood pressure, *PGD* primary glomerular disease

Nephrotic syndrome was the most frequent indication for renal biopsy (2002 patients, 51.7%) followed by AUA (709 patients, 18.3%) and nephritic syndrome, including RPGN (691 patients, 17.8%). Biopsy indications for different pathological diagnoses are presented in Table 1. In more than 80% of patients with MN and MCD, the renal biopsy was performed due to nephrotic syndrome. In addition, nephrotic syndrome was the indication for renal biopsy in more than 60% of those with FSGS and type 1 MPGN. In more than half of the patients with IgAN, AUA and nephritic syndrome, including RPGN, was the indication for renal biopsy.

Renal biopsies were performed in departments of nephrology and radiology in 75.4 and 24.5% of patients, respectively. The mean total number of glomeruli per biopsy was 17 ± 10, and the number of globally sclerotic glomeruli per biopsy was 3 ± 4. The biopsies performed between 1994 and 2019 were included in the study. However, about half of the data were recorded from patients over the period 2013–2019. The pathological diagnoses in patients who had renal biopsy due to nephrotic syndrome were MN, FSGS and MCD, respectively. The most frequent diagnosis was IgAN in patients with AUA.

Examination of urinary sediment revealed microscopic hematuria (> 5 erythrocytes per high power field) in 50.1% (1699 of 3391 and pyuria (> 5 leukocytes per high power field) in 19.2% (645 of 3230) of patients.

### Comparison of the data with 2014 data

**Fig. 4:**
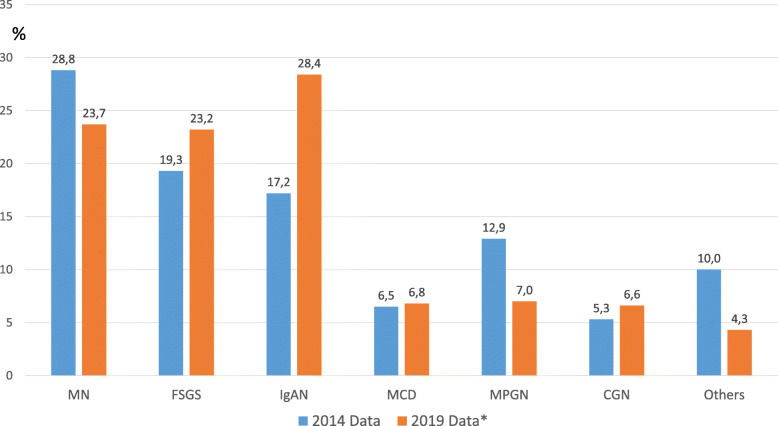
The biopsy diagnoses of the patients compared between the participants involved in the 2014 article [5] and newly diagnosed patients after then. The changes in the rates of all PGDs, except CGN, were statistically significant. *Includes only the patients diagnosed after 2014 manuscript. (Abbreviations: MN: membranous nephropathy, FSGS: Focal and segmental glomerulosclerosis, IgAN: IgA nephropathy, MCD: minimal change disease, MPGN: membranoproliferative glomerulonephritis, CGN: crescentic glomerulonephritis)

**Fig. 5:**
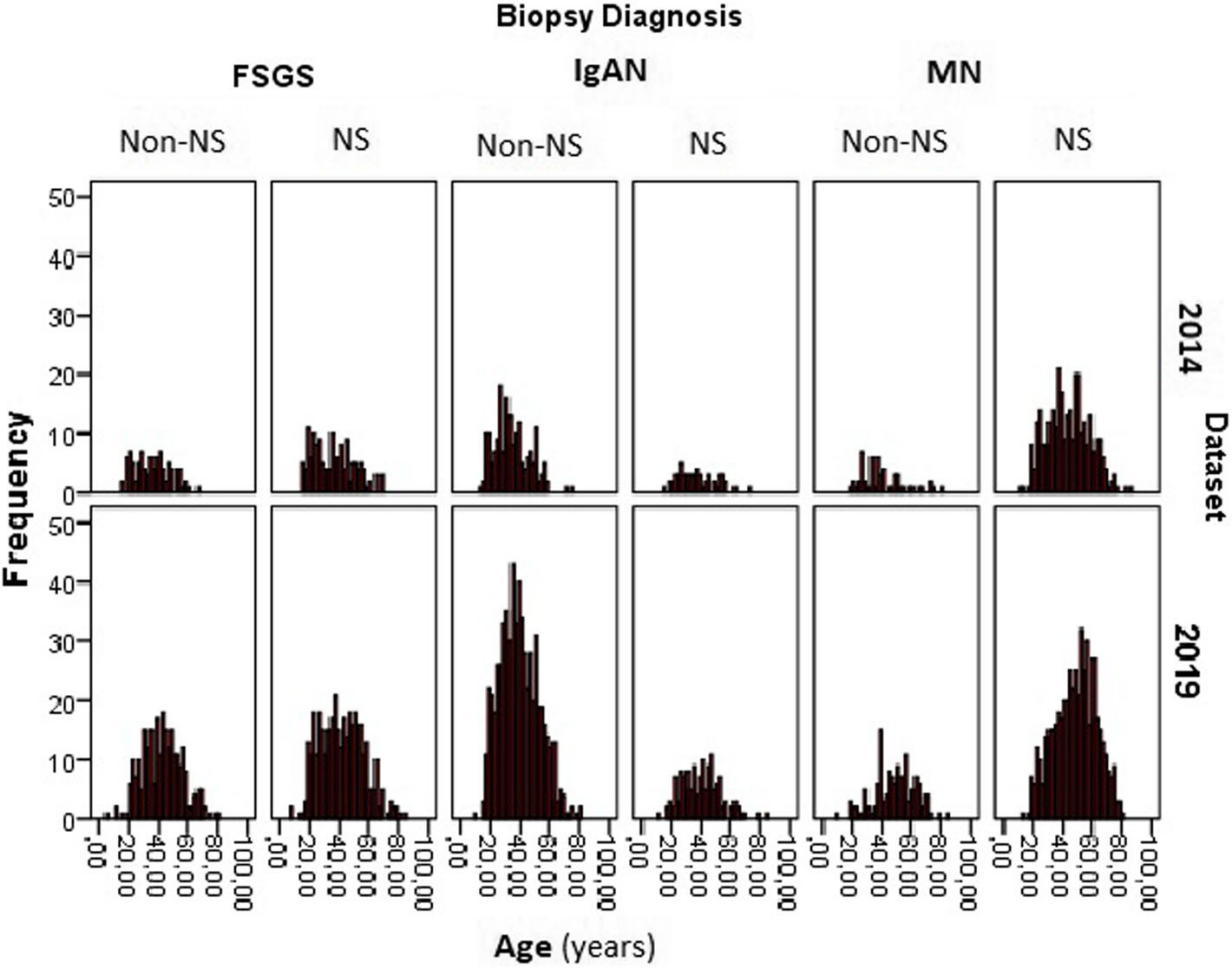
Age distribution curves of major PGDs according to the biopsy period and biopsy indication. 2019 dataset includes only the data of the patients diagnosed after 2014 manuscript. The mean age of all these PGDs increased in 2019 compared to the 2014 database (ages according to the databases 2014 and 2019: IgAN: 35.2 ± 12.2 vs. 39.5 ± 13.1; MN: 43.4 ± 14.5 vs. 48.6 ± 14.1; FSGS: 36.0 ± 13.3 42.1 ± 14.2, respectively).(Abbreviations: MN: membranous nephropathy, FSGS: Focal segmental glomerulosclerosis, IgAN: IgA nephropathy, NS: Nephrotic syndrome)

We compared our patient data in our article published in 2014 [5] with the new patient data added to our dataset from 2014 to 2019. The changes in the rates of PGDs are given in Fig. 4. The changes in the rates of all PGDs, except CGN, were statistically significant. Although nephrotic syndrome continued to be the most common renal biopsy indication, its frequency decreased significantly (57.8% in 2014, 48.7% in 2019). Moreover, the median age of the patients increased significantly [median (interquartile range-IQR): 39 (29–51) years in 2014 vs. 42.5 (32–54) years in 2019, *p* < 0.05]. Figure 5 shows the age distribution curve of most frequently diagnosed PGDs (IgAN, MN, and FSGS) according to biopsy indications. The mean age of all these PGDs increased in 2019 compared to the 2014 database (ages according to the databases 2014 and 2019: IgAN: 35.2 ± 12.2 vs. 39.5 ± 13.1; MN: 43.4 ± 14.5 vs. 48.6 ± 14.1; FSGS: 36.0 ± 13.3 42.1 ± 14.2, respectively). When the percentage of the biopsy indication for each PGD between the time periods was analysed (Table 4), biopsy indication in IgAN due to AUA significantly increased from 25.5% in 2014 to 39.8% in 2019, but NS indication in MN was not considerably changed (88.2% in 2014 and 81.3% in 2019). The average number of total glomeruli and the number of globally sclerotic glomeruli per biopsy were statistically significantly higher in 2019 data when compared to 2014 data [median (IQR): 16 [7–20] vs. 13 [7–17, 21, 22] and 1 (0–4) vs. 1 (0–3), respectively].

## Discussion

In this study, we presented comprehensive, up-to-date data of the multicentre glomerular diseases study performed by the TSN-GOLD Working Group. There were 1274 patients in the first data published in 2014 [5]. The number of cases has increased by approximately threefold (3875 patients) and has reached one of the highest published PGD data. The most significant change between these data is that the frequency of IgAN increased from 17.2 to 25.7%, and become the most common PGD diagnosed via renal biopsy in Turkey. Another important point is that the frequency of biopsy indication due to AUA and nephritic syndrome significantly increased (from 10.8 to 17.8% and from 16.6 to 18.2%, respectively). IgAN was found to be the most common PGD in our patients who underwent kidney biopsy due to AUA (Fig. 1). When these data are combined, it can be interpreted that the increase in the frequency of IgAN in our patients is mainly related to the change in biopsy indications rather than the increase in the frequency of IgAN. As is known, there are significant regional differences in the prevalence of PGDs.

For the comparison of similar international data, we created a table (Table 3) from similar articles reporting the frequency of GN published in different parts of the world. We tried to obtain a more formal table by eliminating the secondary GN causes from the data and create an easily understandable and comparable data. However, as can be seen in this table, both the first publication and present data present biopsy results in patients with nephrotic syndrome in our country; biopsy results are more consistent with European data and MN is detected more frequently. However, IgAN is common in Asian countries and FSGS in the United States.

**Table 3:** Similar data from some of the published primary glomerulonephritis studies generated from international data

Country	All PGD Patients(%)			Patients with nephrotic syndrome			Biopsy indication (%)	
	1st	2nd	3rd	1st	2nd	3rd	Nephrotic syndrome	AUA
Turkey (TSN-GOLD 2014) [5]	MN (28.8%)	FSGS (19.3%)	IgAN (17.2%)	MN (43.2%)	FSGS (19.7%)	MCD (10.3%)	57.8	10.8
Turkey (TSN-GOLD 2019)^a^	IgAN (25.7%)	MN (25.6%)	FSGS (21.9%)	MN (39.8%)	FSGS (25.2%)	MCD (11.0%)	52	18
France [23]^b^	IgAN (29.7%)	MN (19.0%)	CGN (16.8%)	–	–	–		
Italy [24]	IgAN (43.5%)	MN (23.4%)	FSGS (13.1%)	MN (44.1%)	FSGS (16.9%)	MCD (16.7%)	45	51
Spain [25]^c^	–	–	–	MN	MCD	FSGS	35	25
England [26]	IgAN (38.8%)	MN (29.4%)	MCD (9.8%)	–	–	–		
Czech Republic [27]^c^	IgAN (34.5%)	MCD (12.5%)	Mes.PGN(%11.3)	IgAN**	MN	FSGS	42	37
Lithuania [28]	IgAN(34%)	FSGS (13.2%)	MPGN (12.5%)	–	–	–		
Japan [29]	IgAN (~ 50%)	–	–	MCD (40%)	MN (35.6%)	FSGS (13%)	16.5	48^d^
South Korea [30]	IgAN (38.2%)	MCD	MN	MCD (38.5%)	MN (25.7%)	IgAN (11.1%)		
China [31]	IgAN (45.2%)	Mes. PGD (25.6%)	MN (9.9%)	–	–	–		
Brazil £ [32]	FSGS (29.7%)	MN (20.7%)	IgAN (17.8%)	–	–	–	41.5	27.2
U.S.A. [18, 33]^e^	FSGS	IgAN	MN	FSGS	MN	MCD		
Taiwan [34]	IgAN (26%)	FSGS (21.6%)	MN (20.6%)	MN (28.8%)	MCD (28.2%)	FSGS (24.8%)		
Colombia [11]^f^	IgAN (22.6%)	FSGS (20.1%)	MN (14.9%)					
Poland [12]	IgAN (51.2%)	MPGN (19.3)	MN (11.2%)	MN	FSGS	IgAN		

*Abbreviations*: *AUA* Asymptomatic urinary abnormalities, *CGN* crescentic glomerulonephritis, *FSGS* Focal segmental glomerulosclerosis, *IgAN* IgA nephropathy, *MCD* minimal change disease, *Mes.PGN* Mesangioproliferative glomerulonephritis (non-IgA), *MN* membranous nephropathy, *MPGN* membranoproliferative glomerulonephritis, *PGD* primary glomerular disease, *TSN-GOLD* Turkish Society of Nephrology Glomerular Diseases

^a^ Current study; ^b^ cumulative data of different age and periods, ^c^ including pediatrics cases, ^d^ This percentage was presented as the percentage of nephritic syndrome, but asymptomatic urinary abnormalities seem to be involved also. ^e^ These results are the combination of two different studies [9, 10], ^f^ including secondary cases

When all PGD patients were included, IgAN became the most common PGD in our country and the rate became similar to that in other European and Asian countries. Although IgA deposits may be seen in biopsies of individuals without renal disease, these cases generally do not have clinical features of GN [21, 35], and it is known that differences in the indications for biopsy in management practices across countries can affect the incidence and prevalence of diagnosed PGDs [22]. Routine urine screening is performed in some countries such as Japan and Korea. Such applications may allow the detection and biopsy of AUA cases and thus high AUA rates in GN registries. On the other hand, although our data show that the frequency of biopsies due to AUA seems to be increasing (from 10.8 to 17.8%) in our country, it is still relatively low compared to European and Asian countries. For example, the rate of patients undergoing biopsy for AUA is 51% in Italy, 25% in Spain, 37% in the Czech Republic, and 48% in Japan (Table 3).

The prevalence of IgAN is higher in Asian cohorts and Caucasians, whereas FSGS is more common in North America [7–9]. Another finding consistent with these data is that the percentage of patients diagnosed with MN decreased in parallel with the decrease in the proportion of patients undergoing biopsy for nephrotic syndrome indication. There were significant changes in both biopsy indications and age groups between the 2014 and 2019 data, as presented in Figs. 4 and 5. Out data clearly showed that the peak of PGD in our country moved to older age groups. This might suggests aging may cause an increase of IgAN and the decrease of MN. But biopsy indication in IgAN due to AUA significantly increased from 25.5% in 2014 to 39.8% in 2019, and nephrotic syndrome indication in MN was not considerably changed (88.2% in 2014 and 81.3% in 2019) (Table 4). In the last decade, many studies have shown that even proteinuria at the level of 0.5-1 g/day has a significant effect on the prognosis of IgAN [10–12] and steroid treatment may have a positive effect on prognosis in such IgAN patients [13–16]. This may be the most important reason for the recent increase in the frequency of renal biopsy for AUA and, hence IgAN, in our country. In addition, the increase in the number of nephrologists and the number of centres that can perform a kidney biopsy in our country is likely to play an important role in performing biopsy earlier in patients with AUA.

**Table 4:** The percentage of the biopsy indication for each PGD according to the biopsy period

	2014 Data (%)				2019 Data (%)			
	AUA	Mixt NS	Nephritic syndrome	NS	AUA	Mixt NS	Nephritic syndrome	NS
IgAN	25.5	9.5	37.0	28.0	39.8	5.3	35.5	19.4
FSGS	14.7	8.0	13.8	63.6	21.5	4.7	11.1	62.7
MN	5.2	3.8	2.9	88.2	13.7	3.5	1.5	81.3
MCD	2.7	2.7	1.4	93.2	9.0	1.7	2.8	86.4
MPGN	7.3	8.7	15.3	68.7	16.6	5.3	16.6	61.5
CGN	5.1	10.2	71.2	13.6	8.0	9.3	73.5	9.3

*Abbreviations*: *AUA* asymptomatic urinary abnormalities, *NS* Nephrotic syndrome, *IgAN* IgA nephropathy, *FSGS* Focal segmental glomerulosclerosis, *MN* membranous nephropathy, *MCD* minimal change disease, *CGN* crescentic glomerulonephritis

Another important point in our study was the increased prevalence of FSGS in all patient groups, more prominent in patients with nephrotic syndrome (Table 3, Fig. 2). FSGS is the most common cause of nephrotic syndrome in the United States [17–19]. However, there are publications indicating that the incidence of FSGS decreases relatively in biopsy patients due to the increased prevalence of diabetic nephropathy [20]. There are similar publications from Brazil (Table 3). In other regions, FSGS is the second most common cause of nephrotic syndrome after MN. In order to explain the increase in the frequency of FSGS in our data, we need more detailed data. However, compared to our previous report, we found that our rate of patients with diabetes (possibly also with high body mass index) and hypertension has increased (from 6 to 10% and from 26 to 32.4%, respectively) and this might have eventually increased the frequency of secondary FSGS. Therefore, it cannot be ignored that some of these secondary FSGS patients might have been registered as having primary FSGS in our database.

MN was more common in patients over 40 years of age (Fig. 3). This finding is compatible with many European data [24–26]. On the other hand, IgAN was the most common PGD in the group under 40 years of age. This finding is consistent with European and Asian data, as well as the United States and Brazilian data (where FSGS was the most common PGD when all patients were included) [17, 18, 32].

There are some limitations of this study. We included only the data obtained at the time of renal biopsy. As we are currently collecting data on our long-term results, we have not included these data (renal outcomes, treatments, etc.) in this study. Misclassification due to different definition or measurement variables could be a problem in all centres. Since we excluded patients without light microscopy and IF findings, errors in the diagnosis of glomerular diseases such as FSGS, which are likely to have such diagnostic confusion, were minimized. For this purpose, we excluded more than 500 patients. In addition, the identity information and centre information of each patient registered in the database were recorded anonymously in order to minimize the patient selection bias. Another point is, as in every retrospective study, there were patients who lacked some data. Also, although we asked the centers to include all their patients’ data complying with the inclusion criteria, there might have been some patients that were recorded or data that some centres failed to input to the database. The biopsy specimens were examined only by local pathologists and the diagnosis could not be confirmed by another pathologist. However, all these limitations are common problems in most recording systems. The subtypes of FSGS and MPGN were not specified because pathology reports do not routinely include FSGS subtypes, and the new classification of MPGN, according to IF findings, have recently begun to be included in renal biopsy reports in our country. Therefore, we did not present these subgroups data. On the other hand, only PGDs were included in our study, and the database has been specially prepared for it. This is the major advantage of the present study because previous epidemiological studies involved both primary and secondary glomerular diseases. Eventually, we assume that our data has the power to represent PGD patients across the country.

## Conclusions

The distribution of PGDs in Turkey has become similar to that in other European countries. IgAN diagnosed with renal biopsy has become more prevalent compared to MN, becoming the most common PGD in Turkey. MN is still the most common cause of nephrotic syndrome among PGDs.

## Data Availability

We created a database for primary glomerular diseases with the name of ‘Turkish Society of Nephrology Glomerular Diseases Working Group (TSN-GOLD) in 04.04.2008. We published the first results of our study in 2014 and now, we present here the extended results. The data can be found at the following address: http://pgh.tsn.org.tr/login.php

## Abbreviations

Acute PGN: Acute proliferative glomerulonephritis
AUA: Asymptomatic urinary abnormalities
CGN: Crescentic glomerulonephritis
CKD: Chronic kidney disease
CKD-EPI: Chronic kidney disease epidemiology collaboration
ERA-EDTA: European Dialysis and Transplant Association
ESRD: End-stage renal disease
IF: Immunofluorescence microscopy
IgAN: IgA nephropathy
FSGS: focal segmental glomerulosclerosis
GN: Glomerulonephritis
MCD: Minimal change disease
Mes.PGN: Mesangioproliferative glomerulonephritis (non-IgA)
MN: Membranous nephropathy
MPGN: Membranoproliferative glomerulonephritis
PGD: Primary glomerular disease
PGDs: Primary glomerular diseases
TSN: Turkish Society of Nephrology
TSN-GOLD: Turkish Society of Nephrology Glomerular Diseases
USRDS: United States Renal Data System
